# Membrane Transporters of the Major Facilitator Superfamily Are Essential for Long-Term Maintenance of Phenotypic Tolerance to Multiple Antibiotics in E. coli

**DOI:** 10.1128/Spectrum.01846-21

**Published:** 2021-11-17

**Authors:** Yingkun Wan, Miaomiao Wang, Edward Wai Chi Chan, Sheng Chen

**Affiliations:** a Department of Infectious Diseases and Public Health, Jockey Club College of Veterinary Medicine and Life Sciences, City University of Hong Kong, Kowloon, Hong Kong; b State Key Lab of Chemical Biology and Drug Discovery, Department of Applied Biology and Chemical Technology, The Hong Kong Polytechnic University, Kowloon, Hong Kong; University of Guelph

**Keywords:** antibiotic tolerance, membrane transporter, efflux pump, MFS

## Abstract

Antibiotic tolerance is not only the key underlying the cause of recurrent and chronic bacterial infections but it is also a factor linked to exacerbation of diseases, such as tuberculosis, cystic fibrosis-associated lung infection, and candidiasis. This phenomenon was previously attributed to a switch to physiological dormancy in a bacterial subpopulation triggered by environmental signals. However, we recently showed that expression of phenotypic antibiotic tolerance during nutrient starvation is highly dependent on robust production of proteins that actively maintain the bacterial transmembrane proton motive force (PMF), even under a nutrient-deficient environment. To investigate why PMF needs to be maintained for expression of phenotypic antibiotic tolerance, we tested the relative functional role of known transporters and efflux pumps in tolerance development by assessing the effect of deletion of specific efflux pump and transporter-encoding genes on long-term maintenance of antibiotic tolerance in an Escherichia coli population under starvation. We identified eight specific efflux pumps and transporters and two known efflux pump components, namely, ChaA, EmrK, EmrY, SsuA, NhaA, GadC, YdjK, YphD, TolC, and ChaB, that play a key role in tolerance development and maintenance. In particular, deletion of each of the *nhaA* and *chaB* genes is sufficient to totally abolish the tolerance phenotypes during prolonged antimicrobial treatment. These findings therefore depict active, efflux-mediated bacterial tolerance mechanisms and facilitate design of intervention strategies to prevent and treat chronic and recurrent infections due to persistence of antibiotic-tolerant subpopulations in the human body.

**IMPORTANCE** We recently showed that the antibiotic-tolerant subpopulation of bacteria or persisters actively maintain the transmembrane proton motive force (PMF) to survive starvation stress for a prolonged period. This work further shows that the reason why antibiotic persisters need to maintain PMF is that PMF is required to support a range of efflux or transportation functions. Intriguingly, we found that tolerance-maintaining efflux activities were mainly encoded by 10 efflux or transporter genes. Because our study showed that deletion of even one of these genes could cause a significant reduction in tolerance level, we conclude that the products of these genes play an essential role in enhancing the survival fitness of bacteria during starvation or under other adverse environmental conditions. These gene products are therefore excellent targets for future design of antimicrobial agents that eradicate antibiotic tolerant persisters and prevent occurrence of chronic and recurrent human infections.

## INTRODUCTION

In 1944, Joseph Bigger discovered a phenomenon in which bacteria, including nonresistant strains that could not grow in the presence of antibiotics, always contain a subpopulation that could survive treatment of a high concentration of antibiotic (1, 2). This phenomenon has since become known as antibiotic tolerance, and the bacterial subpopulation that resists antimicrobial action may also be termed “persisters.” Since then, persisters have been found in almost all bacterial species and are now known to play an important role in causing recurrent and chronic infections due to their ability to survive antimicrobial treatment and regrow afterwards. Their existence is also believed to exacerbate symptoms of bacterial infection and prolong treatment time.

Cellular mechanisms underlying expression of the tolerance phenotype are different from those of antibiotic resistance; the latter is due to genetic mutations or acquisition of resistance-encoding determinants from exogenous sources. Tolerance is a kind of self-defense yet reversible physiological mechanism that protects the bacterial cell against the deleterious effects of antibiotics, the host immune response, or other adverse conditions, such as starvation. Tolerance is not heritable, which means that regrowth of the subpopulation of antibiotic persisters to a largely nontolerant population may occur when environmental conditions become favorable. In 2013, Wood et al. performed an elegant experiment that demonstrated that persisters and resistant subpopulations coexisted within a bacterial population subjected to antibiotic selection pressure. Within 2 h of antibiotic treatment, most of the bacterial cells were killed, leaving behind only persisters and resistant mutants. Following withdrawal of antibiotic, however, the resistant mutant subpopulation proliferated sharply, whereas the size of the population of nonresistant but drug-tolerant persisters remained unchanged (3). This experiment confirmed that antibiotic-tolerant and resistant subpopulations exhibit different cellular mechanisms that enable bacteria to survive under the bactericidal action of antibiotics.

Persisters may be regarded as a phenotypically heterogeneous, metabolically quiescent bacterial subpopulation that exhibits multidrug tolerance phenotypes. A number of previous studies showed that tolerance formation was mainly associated with cellular dormancy (4–6). However, several physiological pathways that underlie formation of bacterial antibiotic tolerance by mediating onset of dormancy have been discovered (2, 7). Products of specific genes in these pathways play an important role in tolerance formation. An example is the toxin-antitoxin (TA) loci system, in which the TA locus encodes two proteins, a stable toxin that can interrupt essential cellular pathways and induce a dormancy-like state and a labile antitoxin that can conjugate the toxin to nullify such toxicity. Apart from the TA systems, genes in various other pathways are found to be involved in tolerance formation, including the stringent response (8), SOS response (9, 10), energy metabolism (11, 12), global regulators (13), *trans*-translation (14, 15), and various signaling pathways (16). These tolerance-inducing mechanisms mainly involve mediating onset of cell dormancy or metabolic changes, which are regarded as passive mechanisms that confer stress tolerance by shutting down physiological activities; however, it was recently found that persisters actually actively express defense mechanisms, such as efflux, in response to extreme conditions, (17).

Our laboratory recently discovered that the tolerance phenotype inducible by long-term starvation may also be conferred by various actively induced cellular mechanisms, including expression of the phage shock proteins (Psp), which are responsible for maintenance of proton motive force (PMF) (18). PMF is generated when protons are transported across the bacterial cell membrane, resulting in formation of a proton gradient. The electron transport chain, which comprises the redox reaction components, is the machinery that generates the proton motive force by providing the energy required to pump protons out of the bacterial cell membrane. Our preliminary study further showed that maintenance of PMF is essential for bacteria to express phenotypic tolerance to β-lactam antibiotics. However, the underlying mechanisms by which PMF helps maintain phenotypic tolerance in bacteria remain poorly defined. PMF is known to be required for activities of specific membrane proteins, such as efflux pumps and various transportation proteins. It is therefore likely that the need to maintain PMF in tolerant cells is due to the requirement to facilitate proper functioning of specific membrane proteins that play a role in expression of the tolerance phenotype. If tolerant cells are dormant, activities of membrane proteins should be kept at a minimum. However, antibiotic accumulation is known to be one main feature of bacteria tolerance (18). In Gram-negative bacteria, two factors determine the level of intracellular antibiotic accumulation: membrane permeability and efflux activity. Aminoglycosides and macrolides, both hydrophobic antibiotics, gain access into the cell through the membrane by metabolic activity-dependent diffusion. In comparison, β-lactam antibiotics, the most important category of antibiotics used to treat bacterial infections, diffuse passively into bacterial cells via outer membrane porins. Containing a β-lactam ring, the bactericidal effect of these agents is due to their ability to inhibit cell wall synthesis. Our recent finding that PMF is required for long-term maintenance of the antibiotic tolerance phenotype implies that a number of membrane proteins may be actively involved in expression of phenotypic antibiotic tolerance. To explore this idea, we attempted to identify efflux and transportation genes that are highly expressed during short- and long-term starvation and further shortlist membrane proteins whose functions are PMF dependent and play a major role in producing and maintaining a starvation-induced antibiotic tolerance phenotype.

Currently, it is known that bacteria have five major superfamilies of membrane transporters, namely, ATP-binding cassette superfamily (ABC), major facilitator superfamily (MFS), small multidrug resistance family (SMR), resistance-nodulation-cell division superfamily (RND), and multi-antimicrobial extrusion protein family (MATE) (19). Among these, members of the ABC superfamily are the primary transporters that utilize ATP as the driving force of transportation activities, whereas those of the other families are secondary transporters that utilize proton or sodium gradients as energy sources. In this work, we identified a number of efflux pumps and transporters that are actively involved in tolerance formation during starvation. As expected, such efflux pumps and transporters utilize mainly ATP and PMF to drive their transport activities. We found that deletion of genes encoding several efflux pumps led to accumulation of antibiotic in the cytoplasm and hence a sharp drop in the level of tolerance level to various types of antibiotics, especially during long-term treatment, compared to the wild-type strain. These findings confirm that the tolerant subpopulation needs to actively maintain specific efflux activities to express the tolerance phenotype and that suppression of such efflux activities may offer a novel approach to control clinical problems due to bacterial antibiotic tolerance.

## RESULTS

Our preliminary data showed that active maintenance of PMF during starvation is required for prolonged expression of the antibiotic tolerance phenotype and that disruption of PMF would result in rapid resensitization of the tolerant cells to various antibiotics (18). We hypothesize that the reason why the antibiotic-tolerant bacterial subpopulation needs to maintain PMF is that a substantial level of PMF is required for proper functioning of a range of integral membrane proteins that play an active role in mediating prolonged expression of phenotypic tolerance during starvation.

### Efflux pumps and other membrane transporters that are up- and downregulated during starvation tolerance responses.

**FIG 1:**
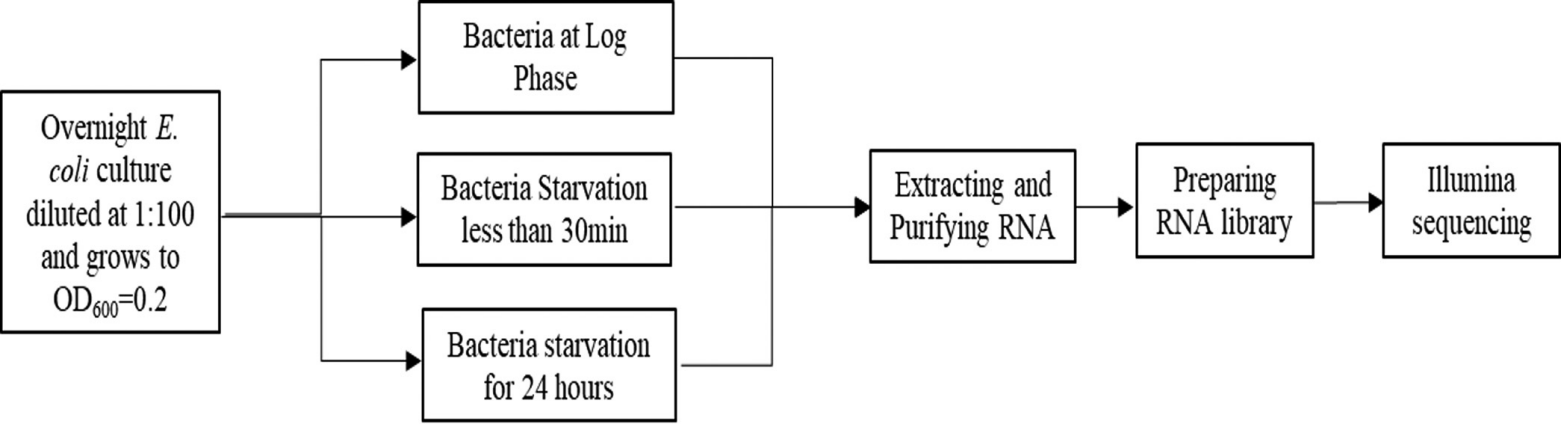
Flowchart illustrating the procedures for preparation of samples for RNA sequencing.

In order to determine whether specific efflux pump- and transporter-encoding genes are actually overexpressed in antibiotic-tolerant cells that have experienced nutrient starvation for 24 h compared with exponentially growing cells, we performed RNA sequencing (Fig. 1) and examined the expression status of these genes. As shown in Table S1 in the supplemental material, 225 of the 321 known efflux and transporter genes tested were found to be overexpressed, whereas only 96 genes were downregulated. This finding is surprising, as metabolic activities in bacteria are expected to have been shut down after encountering nutrient starvation for as long as 24 h. Among the 225 upregulated genes, 136 genes were upregulated more than 4-fold compared to the log-phase population.

### Contribution of upregulated efflux pumps and other membrane transporters to the E. coli starvation tolerance response.

**FIG 2:**
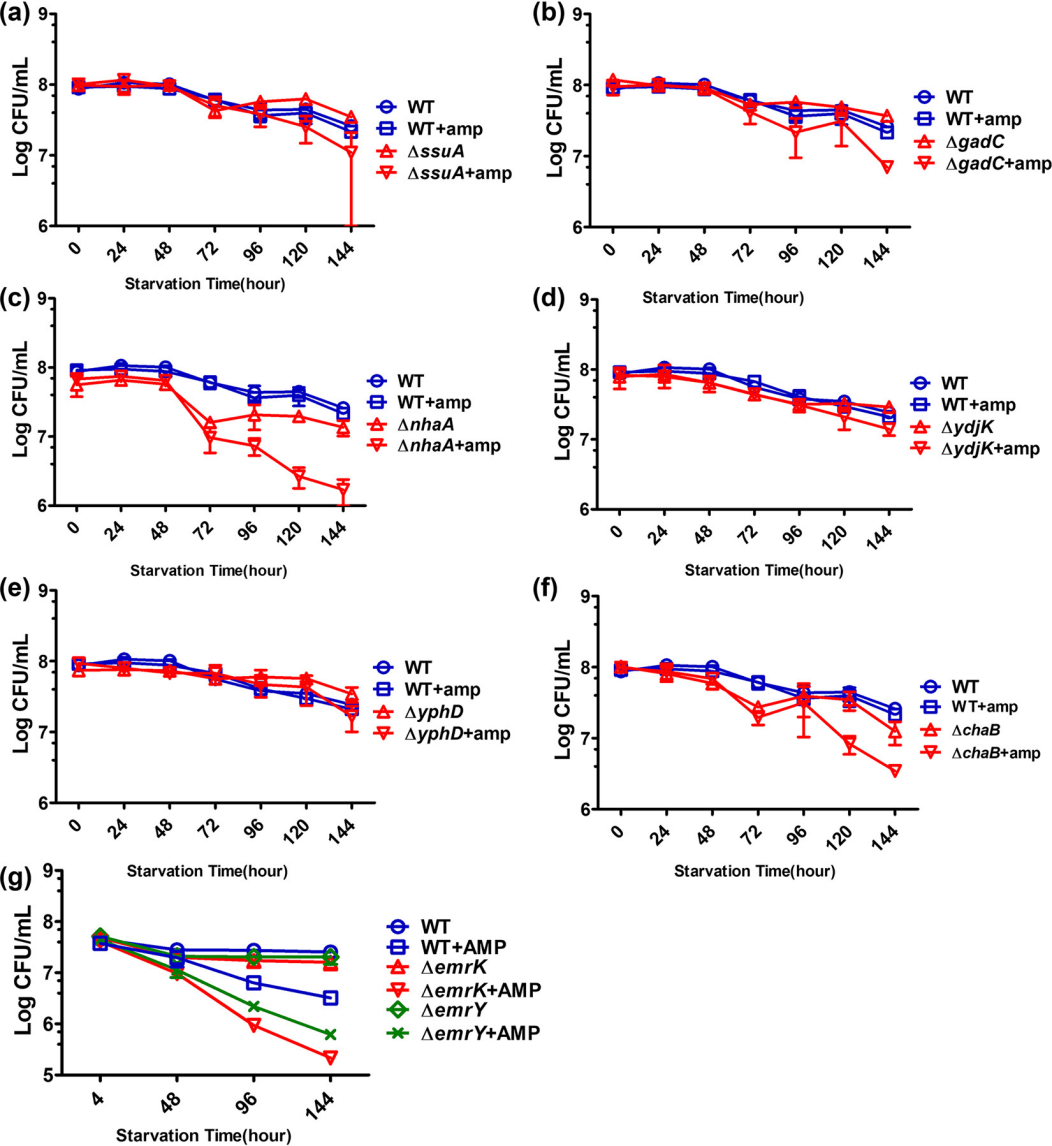
Tolerance levels of deletion mutants of one regulatory and seven transporter genes recorded during a 6-day starvation period. Tolerance levels of *ssuA* (a), *gadC* (b), *nhaA* (c), *ydjK* (d), *yphD* (e), *chaB* (f) and *emrK*, *emrY* (g) knockout strains with or without treatment with 100 μg/ml ampicillin are shown. E. coli strain BW25113 was included as the wild-type control.

The degree of upregulation in gene expression level may not necessarily reflect the relative functional importance of the efflux or transporter genes in tolerance development. We next tested if these starvation-induced efflux and transporter genes indeed played a role in tolerance development by determining the tolerance level of strains in which one or more of these genes whose expression was strongly induced by nutrient starvation were deleted. Among the 136 highly upregulated transporter genes, we successfully created 92 knockout mutants and tested ampicillin tolerance level following starvation for 6 days (144 h). The effect of deletion of a single gene on the ampicillin tolerance phenotype was not apparent, with only a few single-gene knockout mutants exhibiting statistically significant reduction in tolerance level compared with the wild-type strain. Among the 92 knockout strains tested, we found that the tolerance level of eight gene knockout mutants, namely, Δ*emrK*, Δ*emrY*, Δ*ssuA*, Δ*gadC*, Δ*nhaA*, Δ*ydjK*, Δ*yphD*, and Δ*chaB*, decreased significantly compared with the wild-type E. coli strain BW25113. The tolerance levels of other transporter gene knockout strains were similar to that of the wild type, suggesting that these genes do not play an important role in mediating tolerance response in E. coli (Table S2). Among the products of the eight shortlisted genes, the SsuA and YphD proteins are ABC transporters, with SsuA being known to transport sulfonate and YphD being the membrane component of a predicted ATP-dependent sugar transporter. The functions of these transporters are known to be dependent on proton motive force (PMF). On the other hand, GlpT (20) and YdjK belong to the major facilitator superfamily (MFS) of transporters, with GlpT being an important transporter of E. coli with a role in uptake of glycerol-3-phosphate; however, the function of YdjK is not known. NhaA (21) is a sodium ion/proton antiporter that acts to pump sodium ions out of the cytoplasm; this pump is active under alkaline conditions, and its expression is downregulated if the pH is lower than 6.5. ChaB is a putative cation transport regulator whose function is predicted to regulate expression of the sodium-potassium/proton antiporter ChaA (21). EmrK and EmrY also belong to the MFS of transporters, and EmrKY-TolC is an important efflux pump. Among the wide range of membrane proteins tested, TolC is of particular functional importance as it is the central component of a number of bacterial efflux pumps and is responsible for exporting β-lactam antibiotics that have accumulated in the intracellular compartment. High-level expression of the *tolC* gene was recently found to be critical for promoting persister formation (7). TolC has been reported to play an important functional role in various efflux systems, such as the AcrD (22), AcrEF (23), MdsAB (24), and MdtABC (25) pumps, which belong to RND transporters, the MacAB pump (24) of the ABC system, and the MFS transporters EmrAB (21) and EmrKY (23). Despite these findings, there is no systematic and in-depth investigation of the role of membrane proteins in antibiotic tolerance development in bacteria. Therefore, it is necessary to identify the key bacterial efflux pumps or transporters involved in the tolerance development process. However, GadC is a carrier protein that belongs to the APC family and acts to transport gamma-aminobutyric acid (GABA)/glutamate only under acidic conditions (26). Fig. 2 shows that tolerance levels of the *ssuA*-, *ydjK*-, and *yphD*-knockout strains recorded following ampicillin treatment were reduced by 90%, from 10^8^ to 10^7^ CFU/ml in a 6-day treatment period, whereas those of the *emrK*-, *emrY*-, *gadC*-, *nhaA*-, and *chaB*-knockout strains were reduced from 10^8^ to 10^6^ CFU/ml (99% reduction), indicating that these proteins may be involved in tolerance formation. As ChaB is the regulator of MFS transporter ChaA, we also investigated the functional roles of ChaA in formation of phenotypic antibiotic tolerance. The key efflux pump component TolC was also included in subsequent functional analyses.

### Confirmation of the functional role of efflux pumps and other membrane transporters on long-term starvation-induced tolerance response.

**FIG 3:**
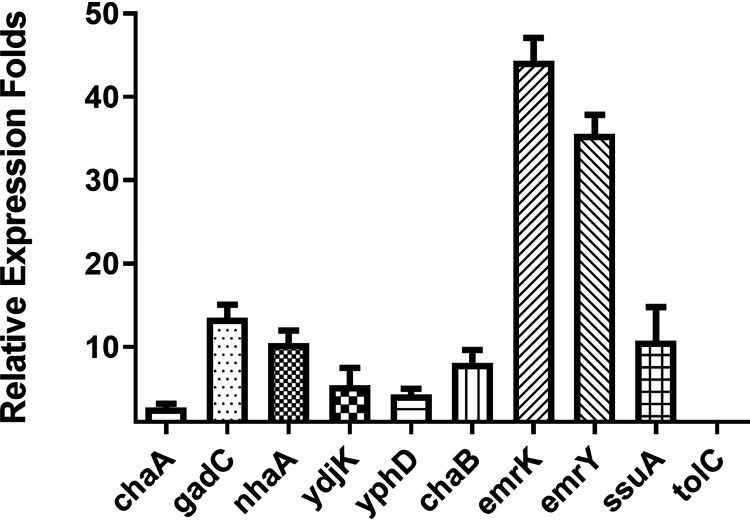
The expression levels of efflux and transporter genes for which the tolerance level decreased significantly in gene knockout mutants. Expression levels of *chaA*, *gadC*, *nhaA*, *ydjK*, *yphD*, *chaB*, *emrK*, *emrY*, *ssuA*, and *tolC* at 24 h with log-phase cells of the wild-type strain BW25113 as a control are shown. The data are expressed as the ratio of gene expression levels of gene knockout mutants and the wild-type strain BW25113.

To investigate whether the 10 shortlisted efflux or transporter genes indeed play a role in expression and maintenance of antibiotic tolerance, we first performed quantitative PCR (qPCR) to confirm the expression level of these genes during nutrient starvation. After encountering starvation for 24 h, expression of the *gadC*, *nhaA*, *emrK*, *emrY*, *ydjK*, *yphD*, *chaB*, *ssuA*, and *tolC* genes was upregulated more than three times compared to the log-phase cells. In particular, expression of the *gadC*, *nhaA*, *emrK*, and *emrY* genes was upregulated as much as 13.3-, 10.3-, 42.5-, and 36.6-fold, respectively (Fig. 3). The qPCR data therefore further confirmed that most of these efflux pumps and membrane transporters are strongly upregulated during starvation-induced tolerance response.

**FIG 4:**
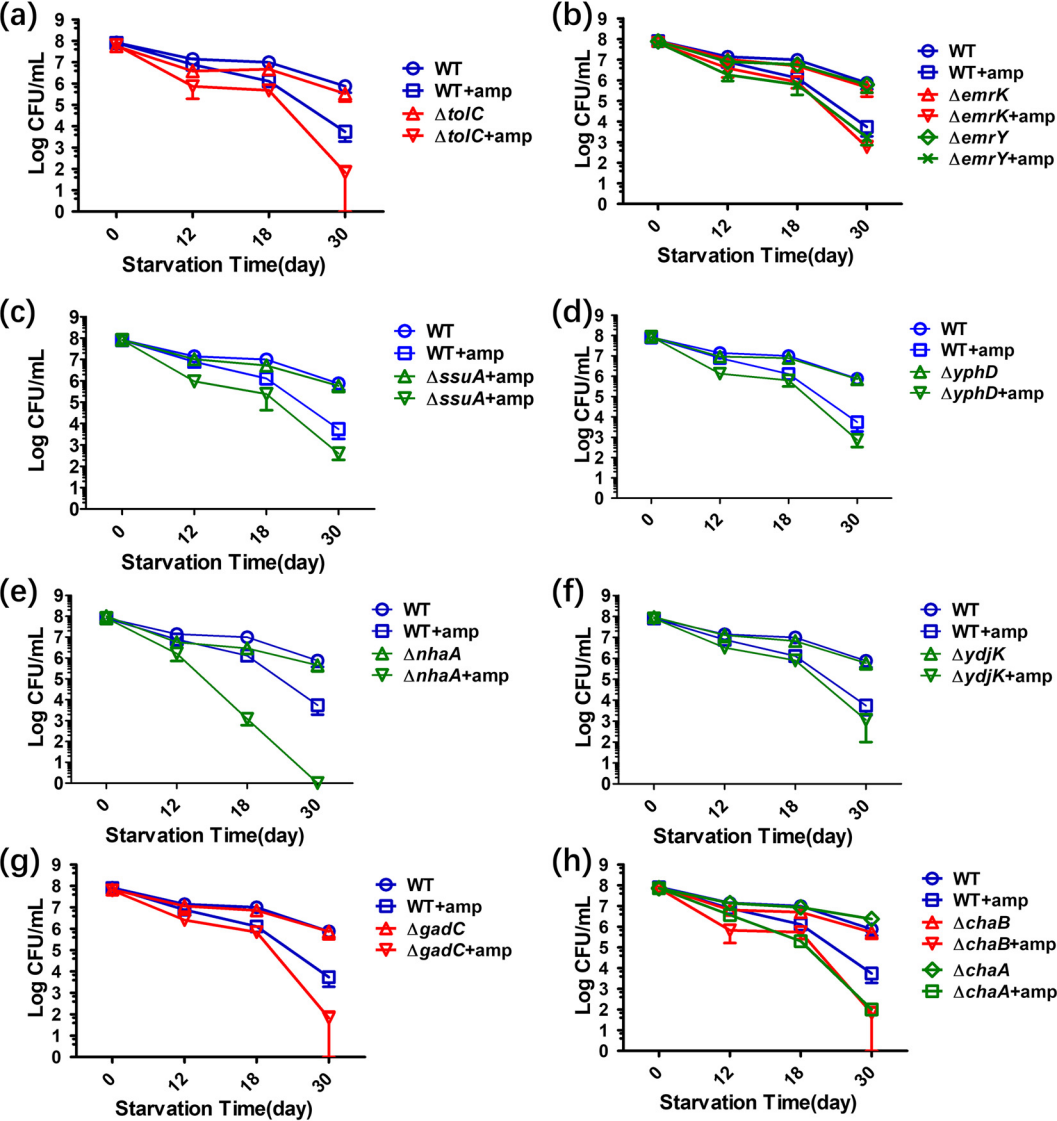
Tolerance level of strains in which specific transporter- and efflux pump component-encoding genes are deleted and then subjected to nutrient starvation for 1 month. The tolerance levels of the *tolC* (a), *emrK*, *emrY* (b), *ssuA* (c), *yphD* (d), *nhaA* (e), *ydjK* (f), *gadC* (g), and *chaB*, *chaA* (h) knockout strains with or without 100 μg/ml ampicillin treatment are shown. E. coli strain BW25113 was included as a wild-type control.

To further examine the effect of deletion of each of the 10 shortlisted genes on the long-term tolerance response, we extended the time frame of the ampicillin tolerance assay to 1 month and determined whether a much larger discrepancy between the level of tolerance of the gene knockout mutants and the wild-type strain could be observed. Following treatment of the starvation population with ampicillin for 1 month, the tolerance level of all the knockout strains tested decreased to a much larger extent compared to the wild type. As shown in Fig. 4, the population sizes of the *tolC*-, *yphD*-, *chaB*-, *chaA*-, and *gadC*-knockout strains dropped from 10^8^ to 10^2^ CFU/ml, whereas that of the *nhaA*-knockout strain shrunk to an undetectable level on day 18, confirming that deletion of specific transporter genes indeed affects long-term maintenance of the tolerance phenotype. It should be stressed that the impact of deletion of a single gene cannot be observed in the short-term (e.g., 24 h) tolerance assay.

We next created double-knockout mutants to determine whether deletion of two efflux/transportation genes resulted in a more significant reduction in tolerance level compared to a single gene knockout strain. As shown in Fig. S1, however, the tolerance levels of the double-knockout strains tested were mostly similar to those of the single-knockout strains in the short-term (6 days) tolerance assay, indicating that the β-lactam tolerance phenotype we observed may be the cumulative effect of activities of multiple efflux pumps and transporters and that the effect of deletion of only one or two genes on tolerance level is not apparent, especially in the short term.

**FIG 5:**
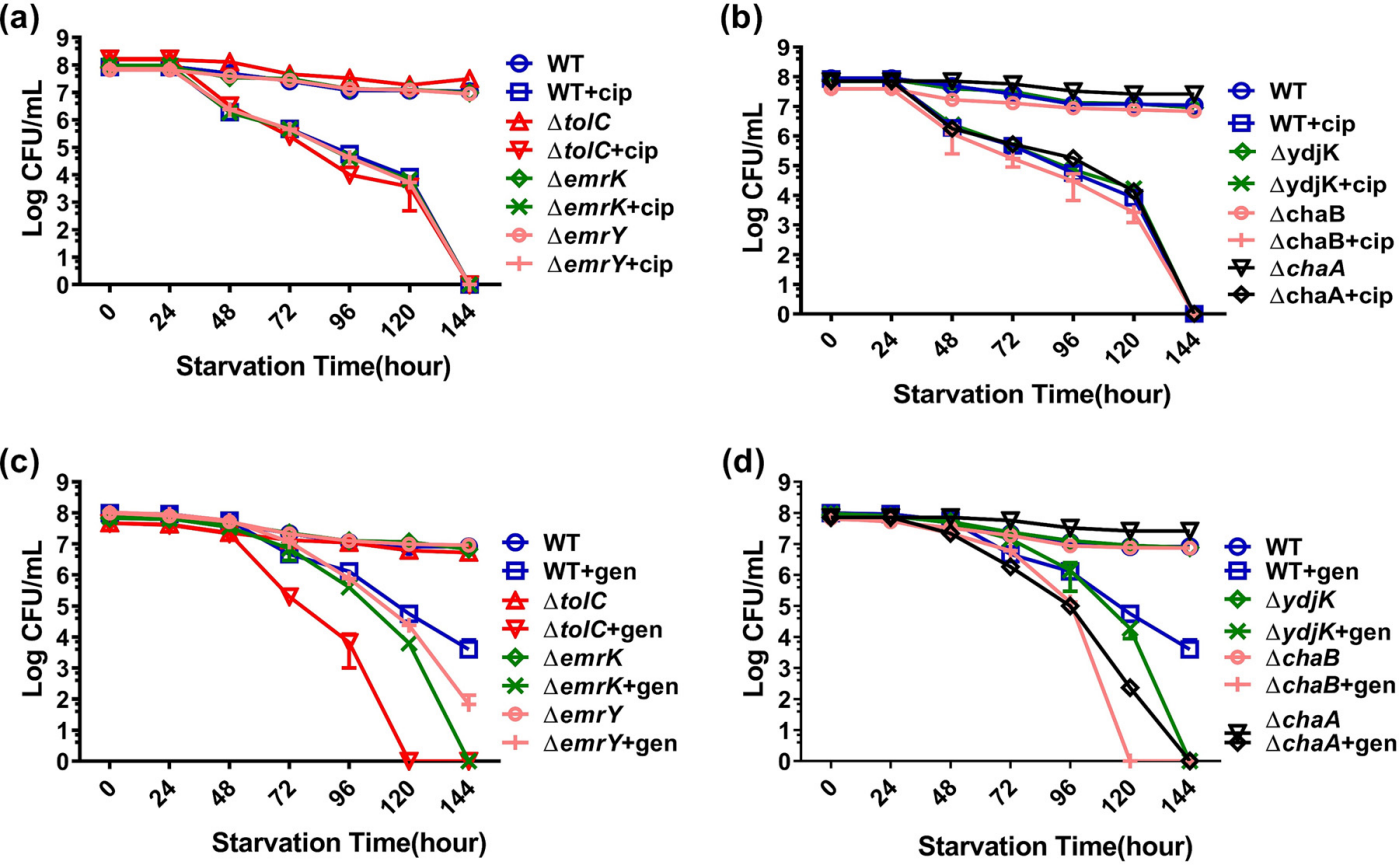
Starvation-induced tolerance to ciprofloxacin and gentamicin in strains in which transporter and efflux pump component-encoding genes are deleted. Tolerance levels of *tolC*, *emrK*, *emrY* (a), and *ydjK*, *chaB*, *chaA* (b) gene knockout strains with or without treatment with 4 μg/ml ciprofloxacin during a 6-day period are shown, and tolerance levels of *tolC*, *emrK*, *emrY* (c) and *ydjK*, *chaB*, *chaA* (d) gene knockout strains with or without treatment with 10 μg/ml gentamicin during a 6-day period are shown. E. coli strain BW25113 was included as a control.

We then tested whether deletion of specific efflux pump- and transporter-encoding genes affected tolerance to other categories of antibiotics. Interestingly, we found that the level of tolerance of the wild-type strain to aminoglycosides and fluoroquinolones was reduced to a much greater extent than that of β-lactams. Deletion of specific transporter genes led to further reduction of the level of tolerance to aminoglycosides but not fluoroquinolones. As shown in Fig. 5, the population size of the *emrK*-knockout strain decreased from 10^8^ to 10^2^ CFU/ml following treatment with gentamicin for 6 days, whereas that of the *chaA*-, *emrY*-, and *ydjK*-knockout strains dropped to an undetectable level. The most significant tolerance suppression effect was recorded in the *tolC*- and *chaB*-knockout strains, the population sizes of which were reduced to undetectable levels on day 5 following exposure to gentamicin. In this work, the concentration of gentamicin and ampicillin used was 10 and 12 times the MIC level of the test strains (10 and 100 μg/ml), respectively. However, the level of tolerance to gentamicin decreased much more significantly in all test strains, including the wild-type strain, than the level of tolerance to ampicillin, indicating that the tolerant cells were much more sensitive to protein synthesis inhibitors than cell wall synthesis inhibitors. We also tested the effect of knockout of other efflux pump-encoding genes whose expression levels were not upregulated under starvation. For the *acrA*- and *acrB*-knockout strains, the population sizes were found to decrease to an undetectable level after 6 days of treatment with gentamicin, whereas that of the wild-type strain only dropped to 10^3^ CFU/ml (Fig. S2). However, ampicillin treatment did not exhibit a significant tolerance suppression effect on these two knockout strains. Nevertheless, these findings appear to suggest that AcrA and AcrB proteins also play a role in maintenance of the tolerance phenotype despite the fact that expression levels of genes encoding these two proteins were not significantly upregulated during starvation (Fig. S1).

### Transportation of antibiotics outside E. coli contributed to the long-term survival of tolerance persisters.

To further confirm the functional role of specific efflux/transportation proteins that were identified in the gene deletion experiments, we tested the level of antibiotic accumulation in specific gene knockout mutants. Antibiotic accumulation is a hallmark of bacterial antibiotic tolerance. Measuring antibiotic accumulation is another approach to sensitively probe the functions of transporter proteins, which may not be accurately reflected by measuring the level of antibiotic tolerance of gene deletion mutants. In Gram-negative bacteria, such as E. coli, the degree of antibiotic accumulation is determined by two factors, membrane permeability and efflux activities. Aminoglycosides and macrolides, which are hydrophobic antibiotics, gain access into the cell through the membrane by membrane potential-driven diffusion. In comparison, hydrophobic β-lactams enter the bacterial cell passively through porin channels (27). Currently, some poorly characterized transporters are known to exhibit efflux functions to reduce antibiotic accumulation in bacteria, leading to onset of antibiotic tolerance (17).

**FIG 6:**
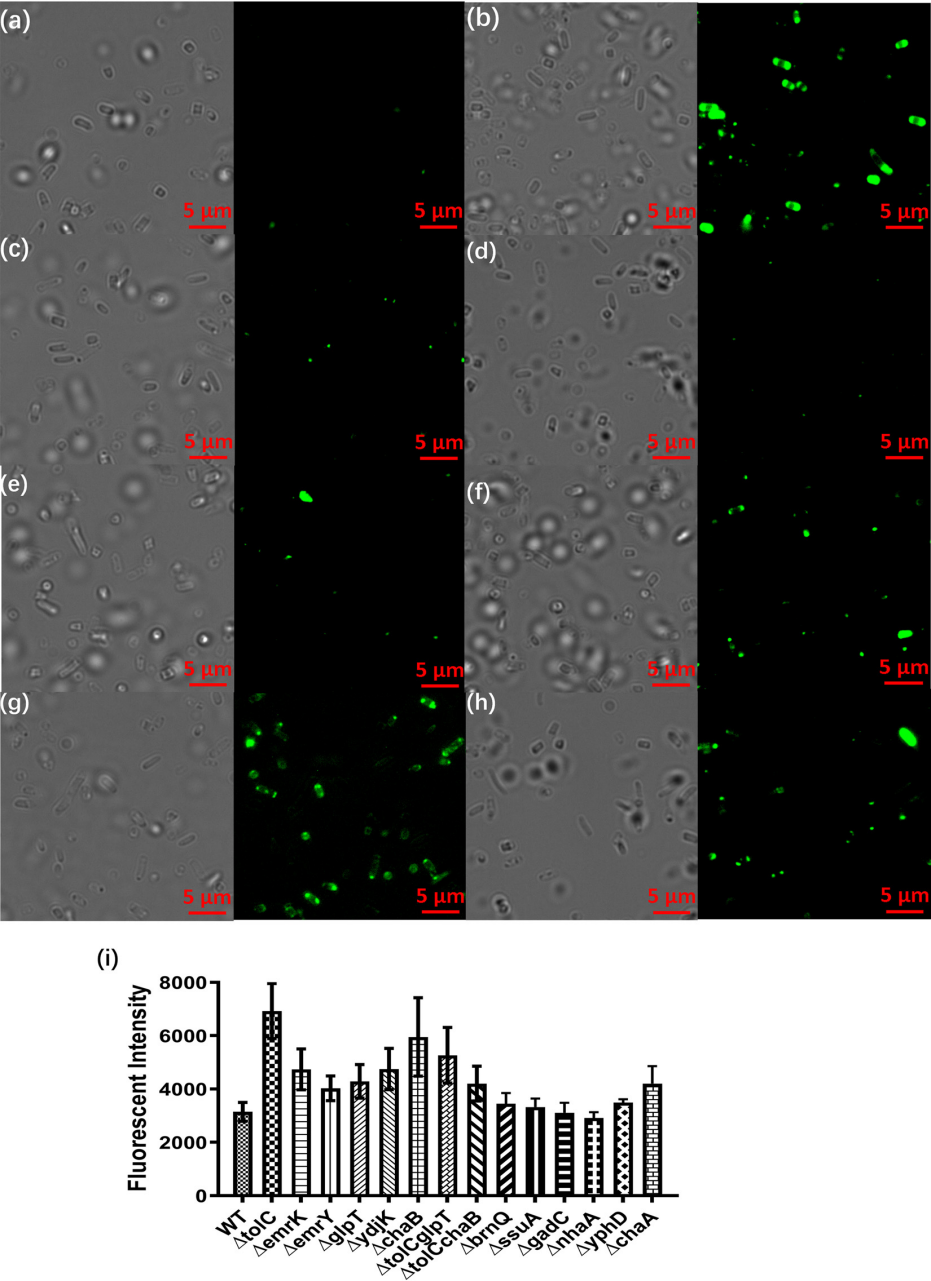
Antibiotic accumulation in strains in which efflux pump- or efflux pump component-encoding genes are deleted. Bocillin accumulation in wild-type (a), *tolC*-knockout strain (b), *emrK*-knockout strain (c), *emrY*-knockout strain (d), *ydjK*-knockout strain (e), *chaA*-knockout strain (f), *chaB*-knockout strain (g), and *tolCchaB* double-knockout strain (h) is shown; left, differential inference contrast (DIC) channel that depicts live images; right, green fluorescent protein (GFP) channel that depicts cells exhibiting fluorescence signal. (i) Fluorescence intensity of wild-type, transporter gene knockout strains, and double-knockout strains recorded after a 6-day starvation period.

The degree of antibiotic accumulation in specific efflux gene knockout strains subjected to starvation for 6 days was measured following treatment with Bocillin FL penicillin. As shown in Fig. 6, Bocillin was almost undetectable in the wild-type strain, but significant accumulation could be detected in the *tolC*-, *chaA*-, and *chaB*-knockout strains. However, only a small amount of Bocillin accumulated in the *emrK*-, *emrY*-, and *ydjK*-knockout strains. The double-knockout strain *tolCchaB* was also subjected to assessment of the degree of antibiotic accumulation (Fig. 5). However, Bocillin was found to accumulate at a lower level in the *tolCchaB* double-knockout strain than in the *tolC* and *chaB* single-gene knockout strains. Nevertheless, both double-knockout strains exhibited a higher level of Bocillin accumulation than the wild-type strain. Findings from the antibiotic accumulation experiments are therefore consistent with those of the gene deletion experiments in that specific transporter genes play an active role in maintenance of phenotypic antibiotic tolerance in bacteria by preventing accumulation of antibiotics in the intracellular compartment.

## DISCUSSION

In a recent work, we showed that the reason why bacterial tolerant subpopulations can resist bactericidal effects of antibiotics is not because they are physiologically dormant; instead, they actively maintain the transmembrane PMF by producing a large amount of the PspA protein under conditions that induce tolerance formation, such as nutrient starvation (18). In this study, we confirmed our hypothesis that bacteria need to maintain PMF during tolerance formation in order to support the functions of a range of membrane proteins, and we identified the range of efflux activities specifically involved in formation and maintenance of phenotypic antibiotic tolerance observable during nutrient starvation. Over 100 known efflux pump- and transporter-encoding genes were found to be overexpressed when the test organisms were subjected to prolonged starvation. This is a surprising finding as it is physiologically costly to produce efflux proteins and generate the energy required to drive efflux activities during nutrient starvation. Among the 10 efflux pumps or efflux pump components that were found to play a key role in tolerance formation and maintenance, five are MFS transporters or components (EmrK, EmrY, YdjK, ChaB, and ChaA). It should be noted that the effect of deletion of genes encoding these transporters was not apparent if the treatment time was short, and even double knockout did not exert a very strong tolerance suppression effect. We originally assumed that this phenomenon was due to the redundant nature of the active tolerance maintenance network, which comprises a large number of efflux and transporter genes, so the effect of deletion of one or two genes would be compensated by the others. However, the tolerance level of a number of gene knockout strains was found to decrease significantly when treatment time was extended to 1 month, whereas the wild-type strain remained highly tolerant to antimicrobial action. This finding not only confirms that efflux activities are required to maintain a tolerance phenotype in the long term but also indicates that each efflux pump is important for the maintenance function, as deletion of one gene may result in total eradication of the tolerant population in the long term. Our works therefore confirm and extend previous findings that showed that efflux activities play an important role in tolerance formation and further show that specific pumps are actually involved in tolerance maintenance, which is a new concept in antibiotic tolerance studies (7, 17).

In the drug accumulation assay, we found that the amount of Bocillin FL penicillin accumulated in the *tolC*-, *chaA*-, and *chaB*-knockout strains was particularly low and much lower than the wild-type strain. We can therefore conclude that the *chaAB* operon plays a particularly important role in tolerance formation, presumably by pumping antibiotics or toxic metabolites out of tolerant cells. In this work, we also found that the starvation-induced tolerant population of the wild-type strain was much more sensitive to fluoroquinolones and aminoglycosides than to β-lactams, indicating that efflux activities elicited during nutrient starvation only confer tolerance to β-lactam but not fluoroquinolones and aminoglycosides. Tolerant cells are supposed to be physiologically dormant, yet this finding indicates that the tolerant cells still undergo active protein and DNA synthesis despite the fact that cell replication does not occur. Apparently, cell wall replacement activities are relatively inactive in tolerant cells compared to protein and DNA synthesis. The role of specific membrane transporters in maintaining phenotypic tolerance to β-lactams during nutrient starvation remains to be elucidated. Among the transport systems that were found to be involved in tolerance maintenance in this work, the activities of ABC transporters are known to be driven by ATP hydrolysis and thus are called primary active transport. Secondary active transport is mediated by members of the major facilitator family and plays a role in transport of nutrients or other substances by utilizing PMF. Transporting one molecule of an electro-neutral substrate by PMF usually consumes one proton, whereas transport by ABC transporters needs two ATP molecules, which are equivalent to six protons. Therefore, MFS transporters are more energy efficient than other kinds of transporters, especially under nutrient-limiting conditions (21). This concept is consistent with our findings that MFS transporters play a more important role in tolerance formation than other types of transporters. To conclude, our works confirm that bacteria actively express specific membrane transport proteins to maintain phenotypic tolerance to antibiotics, especially the β-lactams, and possibly other environmental stresses. Identification of key tolerance-conferring membrane transporters allows development of novel strategies to eradicate physiologically dormant, antibiotic-tolerant bacterial persisters in the human body, thereby greatly reducing the chance of occurrence of recurrent and chronic infections in immunocompromised patients.

## MATERIALS AND METHODS

### Materials.

In this work, E. coli strain BW25113 (28) was used as a wild-type control strain from which specific gene knockout strains were created for functional studies. LB broth and LB agar were used in all bacterial cultures, except that Mueller-Hinton (MH) broth (Hopebio Company) was used in MIC tests. Saline (0.85%) was used to create a starvation-induced antibiotic-tolerant population. l-Arabinose (Sigma) was used in preparation of competent cells. Bocillin FL penicillin (Thermo Fisher) was used as a dye in fluorescence microscopy assays. All antibiotics (ampicillin, gentamicin, ciprofloxacin, and chloramphenicol) were purchased from Sigma. All other single-knockout strains were obtained from the Keio Collection.

### RNA sequencing.

RNA sequencing was performed to measure the expression levels of specific efflux or transporter genes. Genes whose expression levels were upregulated were selected for further studies on the mechanism of tolerance. Briefly, three cultures of the E. coli strain BW25113 were created by inoculating fresh colonies into LB broth followed by incubation at 37°C with shaking until the cell density reached an optical density at 600 nm (OD_600_) of 0.2. Two of the cultures were washed twice with saline, one of which was incubated for 24 h to induce starvation stress. RNA was extracted from the log-phase population and one of the starvation populations by the RNAprotect bacteria reagent and RNeasy protect bacteria kit. The TURBO DNA-free kit was used to completely digest the DNA, and the Ribominus transcriptome isolation kit was used to purify RNA. The NEBNext Ultra II directional RNA library prep kit was then used for preparation of an RNA library for Illumina sequencing. The third culture was also subjected to tolerance assay so that combined analysis of the tolerance level and RNA profile of the starvation and mid-log-phase populations could be performed. Fig. 1 is a flowchart illustrating the procedures for preparation of samples for RNA sequencing.

### Quantitative PCR validation assay.

To validate the expression levels of genes that were found to be highly overexpressed during starvation in the RNA-sequencing experiment, a bacterial culture was treated and divided into three groups, as mentioned above, followed by RNA extraction, purification, and conversion into cDNA via reverse transcription. The cDNA of each of the three samples was then subjected to qPCR to confirm the expression level of the test genes.

### Starvation-induced tolerance assay.

Bacterial cultures at mid-log phase were subjected to centrifugation (6, 000 × *g*, 5 min), followed by removal of supernatant and resuspension of the pellet in 0.85% saline. Antibiotic (ampicillin [100 μg/ml], gentamicin [10 μg/ml], and ciprofloxacin [4 μg/ml]) was added to the cell suspension and then every other day for up to 30 days. All test cultures were incubated at 37°C with shaking at 250 rpm. The survival rate of organisms in the test cultures was calculated by recording the CFU of the culture daily and comparing to that of the original population. Every assay was repeated three times.

### Creation of double gene knockout strains.

The plasmid pKD3, which carries the selectable antibiotic chloramphenicol resistance (*cat*) gene flanked by FLP recognition target (FRT) sites, was used in double gene knockout experiments in which the gene to be deleted was replaced by the *cat* gene (29). Briefly, competent cells of a single-gene knockout strain were prepared by incubating at 37°C until reaching an OD_600_ of 0.3 to 0.5, followed by washing three times with 10% glycerol at 4°C. The plasmid pKD46, which contains the ampicillin resistance gene, was transformed into competent cells, followed by selection of ampicillin-resistant transformants on agar plates containing 100 μg/ml ampicillin. In the next step, competent cells of strains carrying pKD46 were prepared by incubating the strain at 30°C until reaching an OD_600_ of 0.3 to 0.4, followed by addition of 0.5% arabinose, incubation for 1 h, and washing in glycerol three times at 4°C. Finally, the homologous sequence associated with the FRT-flanked chloramphenicol resistance gene was transformed into the competent cells. The double gene knockout strains were selected on agar plates containing 50 μg/ml chloramphenicol.

### Fluorescence microscopy.

An inverted microscope was used for visualization of the structural changes of antibiotic-tolerant persisters during various types of treatment using different laser wavelengths. For measurement of Bocillin accumulation, excitation and emission wavelengths of 488 ± 10 nm and 512 ± 10 nm, respectively, were used. An electron-multiplying charge-coupled-device (EMCCD) camera was used to record the fluorescence signal. The images were analyzed using ImageJ software (Fiji). Antibiotic accumulation of the gene knockout strains that had been subjected to nutrient starvation for 6 days was observed under the fluorescence microscope following treatment with Bocillin FL penicillin. Briefly, 20 g/ml Bocillin FL penicillin was added to the bacterial suspension followed by treatment for 30 min; 0.85% saline was then used to wash out Bocillin FL penicillin to provide a clear background for fluorescence microscopy. The fluorescence intensity was also recorded and subjected to comparison between different samples.
