# Supplementary material for: Membrane Transporters of the Major Facilitator Superfamily Are Essential for Long-Term Maintenance of Phenotypic Tolerance to Multiple Antibiotics in E. coli
Source: Microbiol Spectr. 2021 Nov 17;9(3):e01846-21. doi: 10.1128/Spectrum.01846-21 (PMC8597633; doi:10.1128/Spectrum.01846-21)
Supplement: SUPPLEMENTAL FILE 1 — Supplemental material. Download Spectrum.01846-21-s0001.pdf, PDF file, 0.6 MB [file spectrum.01846-21-s0001.pdf]

Supplementary Materials

# Membrane transporters of the major facilitator superfamily are essential for long term maintenance of phenotypic tolerance to multiple antibiotics in *E. coli*

Yingkun Wan[1#], Miaomiao Wang[1#], Edward Wai Chi Chan[1,2], Sheng Chen[1]*

[1]Department of Infectious Diseases and Public Health, Jockey Club College of Veterinary Medicine and Life Sciences, City University of Hong Kong, Kowloon, Hong Kong;

[2]State Key Lab of Chemical Biology and Drug Discovery, Department of Applied Biology and Chemical Technology, The Hong Kong Polytechnic University, Hung Hom, Kowloon, Hong Kong;

# contribute equally to the work.

*Corresponding author: Sheng CHEN, Email: shechen@cityu.edu.hk;

**Key words:** Antibiotic tolerance, Membrane transporter, Efflux pump, MFS

**Supplementary Table S1. Membrane proteins in *E. coli* strain BW25113 under 24h starvation that were up-regulated or down-regulated at least two folds when compared to the log phase population.**

| Gene | Log2ratio(starvation24h/0h) | Gene | Log2ratio(starvation24h/0h) | Gene | Log2ratio(starvation24h/0h) |
|---|---|---|---|---|---|
| *ugpA,ugpE* | inf | *chaA* | 2.49566 | *znuB,znuC* | 0.426327 |
| *potH,potI* | inf | *afuC* | 2.48727 | *shiA* | 0.409144 |
| *yfdV* | inf | *yqjA* | 2.47646 | *ybaL* | 0.338682 |
| *yeaV* | inf | *clcA* | 2.46971 | *ynjB,ynjC,ynjD* | 0.331064 |
| *ycjO,ycjP* | inf | *yjeM* | 2.4649 | *mlaF* | 0.268055 |
| *yphF* | inf | *ddpB,ddpC,ddpD,ddpF* | 2.46412 | *mdlA,mdlB* | 0.260871 |
| *yddA* | inf | *trkG* | 2.37385 | *flu* | 0.152933 |
| *ydiN* | inf | *phnL,phnM,phnN,phnO* | 2.33477 | *gsiC* | 0.124479 |
| *ydiM* | inf | *yhjX* | 2.33153 | *feoA* | 0.090246 |
| *yaaU* | inf | *yegT,yegU,yegV* | 2.29308 | *oppA* | 0.056681 |
| *ydjK* | inf | *dppB* | 2.26813 | *tyrR* | 0.026136 |
| *dctR* | inf | *ycjN* | 2.25143 | *phnD* | 0 |
| *frlA* | inf | *amtB* | 2.24901 | *yaaJ* | -0.0009 |
| *setC* | inf | *gsiA,iaaA* | 2.24023 | *yicL* | -0.00109 |

| | | | | | |
|---|---|---|---|---|---|
| *ybbW* | inf | *csgG* | 2.23212 | *sbmA* | -0.01483 |
| *ydcT* | inf | *thiB,thiP,thiQ* | 2.21808 | *metQ* | -0.0322 |
| *phnC* | inf | *cycA* | 2.21067 | *ydgI* | -0.03657 |
| *narU* | inf | *yhdZ* | 2.19364 | *yajR* | -0.03773 |
| *mglC* | inf | *hisM,hisQ* | 2.16056 | *yjbB* | -0.07157 |
| *fepB* | inf | *ynfM* | 2.13232 | *ydeA* | -0.09275 |
| *xylF* | inf | *yejF* | 2.11124 | *sapF* | -0.12321 |
| *dppC* | inf | *ydeE* | 2.11084 | *tamA,tamB* | -0.14868 |
| *ddpA* | inf | *yphD* | 2.0923 | *trkA* | -0.17077 |
| *chaB* | inf | *mdtF* | 2.089 | *corA* | -0.21594 |
| *ssuA,ssuD,ssuE* | inf | *yhjE* | 2.07508 | *yhbE* | -0.26187 |
| *yiaM* | inf | *ybaE* | 2.06836 | *yicJ* | -0.26996 |
| *osmY* | 7.50037 | *dauA* | 2.0657 | *sbp* | -0.29457 |
| *mglB* | 7.45462 | *ybbY* | 2.06113 | *gsiD* | -0.30458 |
| *ugpB* | 7.08737 | *btuD,btuE* | 2.05201 | *fucP* | -0.32735 |
| *ybhR* | 6.98614 | *yehX,yehY* | 1.9841 | *ydhC* | -0.331 |
| *emrK,emrY* | 6.4714 | *cysA,cysP,cysU,cysW* | 1.98132 | *yijE* | -0.36438 |

| | | | | | |
|---|---|---|---|---|---|
| *ypjA* | 6.36283 | *yncD* | 1.9599 | *modF* | -0.38292 |
| *cirA* | 6.35824 | *hisJ* | 1.953 | *mlaB,mlaC* | -0.39918 |
| *mntH* | 6.30322 | *yhiD* | 1.94114 | *argP* | -0.4039 |
| *mtr* | 6.03733 | *yhdY* | 1.92931 | *brnQ* | -0.44931 |
| *fhuE* | 5.66645 | *macA,macB* | 1.92631 | *fecB,fecC,fecD* | -0.45319 |
| *mdtI,mdtJ* | 5.5798 | *malG* | 1.91356 | *artI* | -0.50455 |
| | | | | *acrA* | -0.595405 |
| *psuT* | 5.53166 | *yhhS* | 1.90693 | *pheP* | -0.61996 |
| *ytfQ* | 5.50896 | *yojI* | 1.89975 | *yadG,yadH* | -0.62266 |
| *ydcS* | 5.30557 | *alsB* | 1.87919 | *yhfK* | -0.66869 |
| *livK* | 5.16548 | *mppA* | 1.86456 | *lptB* | -0.69155 |
| *exuT* | 5.14076 | *caiT* | 1.83278 | *lysP* | -0.69711 |
| *yphE* | 5.12841 | *ygjI* | 1.81056 | *malF* | -0.79078 |
| *osmF* | 5.11128 | *mhpT* | 1.80754 | *yfbS* | -0.84091 |
| *ygiS* | 5.05624 | *glcA* | 1.79774 | *glnH* | -0.8941 |
| *tonB* | 4.9924 | *yihN* | 1.79009 | *feoB,feoC* | -1.00655 |
| *gntP* | 4.91512 | *hcaT* | 1.73915 | *tnaB* | -1.03225 |

| | | | | | |
|---|---|---|---|---|---|
| *fepA* | 4.91194 | *yjfF,ytfT* | 1.70643 | *trkH* | -1.04073 |
| *argT* | 4.84665 | *gltJ,gltK,gltL* | 1.70302 | *yfeH* | -1.05114 |
| *mglA* | 4.67405 | *potG* | 1.6955 | *rsxG* | -1.13261 |
| *gabP* | 4.60942 | *tauA* | 1.63083 | *znuA* | -1.13309 |
| *ybaT* | 4.49788 | *garP* | 1.60946 | *fecE* | -1.15101 |
| *yhjY* | 4.47765 | *dppD,dppF* | 1.57632 | *cydC* | -1.16341 |
| *ybhI* | 4.35921 | *yehW* | 1.56078 | *glnP,glnQ* | -1.22091 |
| *uhpT* | 4.34496 | *betT* | 1.55969 | *tsgA* | -1.31795 |
| *bssR* | 4.26303 | *gudP* | 1.53729 | *mlaA* | -1.32846 |
| *ydjE* | 4.19745 | *dsdX* | 1.50346 | *fadL* | -1.33607 |
| *fhuF* | 4.18386 | *ugpC,ugpQ* | 1.46291 | *cydD* | -1.33812 |
| *aroP* | 4.05779 | *dppA* | 1.45935 | *modA,modB* | -1.34056 |
| *pnuC* | 4.03155 | *xylG,xylH* | 1.45172 | *yfcJ* | -1.35012 |
| *xylE* | 3.95843 | *fieF* | 1.43449 | *nepI* | -1.3905 |
| *chaC* | 3.89947 | *btuC* | 1.43336 | *btuB,murI* | -1.5071 |
| *yjiJ* | 3.70069 | *yccS* | 1.42152 | *modE* | -1.62543 |
| *yhdX* | 3.64377 | *kdgT* | 1.39655 | *mgtA* | -1.62995 |

| | | | | | |
|---|---|---|---|---|---|
| *yqcE* | 3.64024 | *arsB* | 1.3957 | *rbsA* | -1.79882 |
| *araG* | 3.51306 | *fhuB,fhuC,fhuD* | 1.39337 | *gntT* | -1.81182 |
| *nhaA* | 3.4375 | *nanT* | 1.38972 | *ccmA,ccmB* | -1.81336 |
| *livJ* | 3.40832 | *yebQ* | 1.38434 | *oppB* | -1.83707 |
| *ydcU,ydcV* | 3.40195 | *yidE* | 1.35135 | *artJ* | -1.84593 |
| *tqsA* | 3.38582 | *clcB* | 1.33949 | *btuF,mtn* | -1.9205 |
| *ydcO* | 3.37137 | *livG,livH,livM* | 1.33028 | *potB,potC,potD* | -1.98831 |
| *xapB* | 3.34567 | *ybhL* | 1.31029 | *proP* | -1.99414 |
| *ytfF* | 3.30648 | *zupT* | 1.29744 | *rsxD* | -2.0006 |
| *dcuD* | 3.3 | *tyrP* | 1.26887 | *ccmC,ccmD,ccmE,ccmF,ccmG,ccmH* | -2.00859 |
| *potF* | 3.29315 | *pstA,pstC* | 1.22306 | *dcuB* | -2.08066 |
| *dgoT* | 3.26964 | *mdtE* | 1.21613 | *nupG* | -2.09258 |
| *ssuB,ssuC* | 3.26852 | *yejB,yejE* | 1.11921 | *pstB* | -2.12476 |
| *mdtK* | 3.26287 | *pstS* | 1.11868 | *ykgF,ykgG* | -2.1468 |
| *ygbN* | 3.22012 | *bcr* | 1.10137 | *ybeX* | -2.17132 |
| *ybbA,ybbP* | 3.16664 | *gltS* | 1.0931 | *nikA,nikB,nikC,nikD,nikE* | -2.36868 |
| *yjhF* | 3.16154 | *ydhP* | 1.00458 | *rbsB* | -2.47561 |

| | | | | | |
|---|---|---|---|---|---|
| *ycaM* | 3.14608 | *metI,metN* | 0.989698 | *kup* | -2.50845 |
| *pitB* | 3.14562 | *yhjV* | 0.900552 | *yifK* | -2.56737 |
| *gadC* | 3.05728 | *yagG* | 0.894928 | *gntU* | -2.77355 |
| *ycaD* | 3.04789 | *gsiB* | 0.88291 | *potA* | -2.85724 |
| *yhhT* | 3.02537 | *hisP* | 0.875829 | *oppC* | -2.89236 |
| *cadB* | 2.94417 | *ydhQ* | 0.871154 | *tolC* | -2.904 |
| *tauB,tauC,tauD* | 2.93911 | *tehA,tehB* | 0.867274 | *dcuC* | -3.06691 |
| *fhuA* | 2.93254 | *yejA* | 0.858109 | *proV,proW* | -3.07714 |
| *kgtP* | 2.90104 | *efeO* | 0.84986 | *ykgE* | -3.10226 |
| *cstA* | 2.84223 | *livF* | 0.816854 | *pitA* | -3.11446 |
| *ytfR* | 2.79344 | *yqeG* | 0.800912 | *galP* | -3.19133 |
| *focB* | 2.77477 | *satP* | 0.756376 | *oppD,oppF* | -3.21488 |
| *copA* | 2.76857 | *cysZ* | 0.747703 | *proX* | -3.24109 |
| *araE* | 2.75314 | *yghB* | 0.744645 | *rbsC* | -3.36436 |
| *ybjL* | 2.72977 | *argO* | 0.695129 | *yedE,yedF* | -3.44586 |
| *dctA* | 2.68009 | *fetA,fetB* | 0.693121 | *fecA* | -3.47061 |
| *idnT* | 2.66934 | *actP,yjcH* | 0.684443 | *tdcC* | -3.60234 |

| | | | | | |
|---|---|---|---|---|---|
| *araF* | 2.66167 | *modC* | 0.684118 | *yfcC* | -3.87505 |
| *abgT* | 2.64787 | *mlaE* | 0.677122 | *malE* | -4.17666 |
| *fepC,fepD,fepG* | 2.64679 | *ansP* | 0.650313 | *nupC* | -4.29639 |
| *yiaN* | 2.60395 | *yjeH* | 0.607505 | *malK* | -4.3275 |
| *yeeA* | 2.58737 | *rsxA,rsxB,rsxC* | 0.559082 | *ampG* | -4.9502 |
| *araJ* | 2.54179 | *sapA,sapB,sapC,sapD* | 0.543819 | *sdaC* | -5.3507 |
| *araH* | 2.53344 | *artM,artQ* | 0.534388 | *nirC* | -6.13337 |
| *mdtH* | 2.52607 | *alsA,alsC* | 0.472898 | *glpT* | -6.26243 |
| *cynX* | 2.51828 | *artP* | 0.471682 | *narK* | -6.97498 |

**Supplementary Table S2. Tolerance levels in 8 of the 92 transporter gene knockout strains decreased to at least 90% under 6-day-starvation.**

| Gene knockout strains | Tolerance level decreased (by more than 10%) under 6-day-starvation |
|---|---|
| *eamA* | NO |
| *kefG* | NO |
| *tauC* | NO |
| *ydeE* | NO |
| *ynfM* | NO |
| *ydhP* | NO |
| *betT* | NO |
| *cysP* | NO |
| *nanT* | NO |
| *yjhB* | NO |
| *yeaN* | NO |
| *cysW* | NO |
| *livK* | NO |
| *ugpB* | NO |
| *ugpE* | NO |
| *uhpT* | NO |
| *csgF* | NO |
| *cusF* | NO |
| *gspA* | NO |
| *lsrD* | NO |
| *ycaD* | NO |
| *ydcU* | NO |
| *ydiN* | NO |
| *yphF* | NO |
| *argT* | NO |
| *chaC* | NO |
| *kdpB* | NO |
| *potG* | NO |
| *ssuC* | NO |
| *ycaM* | NO |
| *ydfJ* | NO |
| *ydjK* | YES |
| *ygfS* | NO |
| *yghF* | NO |
| *yhdW* | NO |
| *yiaN* | NO |
| *yicO* | NO |
| *yjeM* | NO |
| *yjhE* | NO |

| | |
|---|---|
| *ytfR* | NO |
| *ytfT* | NO |
| *brnQ* | YES |
| *chaB* | YES |
| *gadC* | YES |
| *nhaA* | YES |
| *phnK* | NO |
| *pnuC* | NO |
| *potF* | NO |
| *potI* | NO |
| *ssuA* | YES |
| *yaaU* | NO |
| *ybaT* | NO |
| *ycjV* | NO |
| *ydcV* | NO |
| *yhjE* | NO |
| *yiaM* | NO |
| *yiaV* | NO |
| *yphE* | NO |
| *ytfQ* | NO |
| *mdtJ* | NO |
| *yhbE* | NO |
| *ycjP* | NO |
| *ybiT* | NO |
| *potC* | NO |
| *nikE* | NO |
| *nirC* | NO |
| *mdtI* | NO |
| *glpF* | NO |
| *kdpA* | NO |
| *kdpC* | NO |
| *mntH* | NO |
| *mtr* | NO |
| *ybiR* | NO |
| *araH* | NO |
| *cysU* | NO |
| *livJ* | NO |
| *ugpA* | NO |
| *cusC* | NO |
| *lsrB* | NO |
| *lsrC* | NO |
| *lacY* | NO |
| *phnE* | NO |
| *yiaO* | NO |

| | |
|---|---|
| *yphD* | YES |
| *ycjN* | NO |
| *glpT* | YES |
| *nikE* | NO |
| *potB* | NO |
| *modF* | NO |
| *emrK* | YES |
| *emrY* | YES |

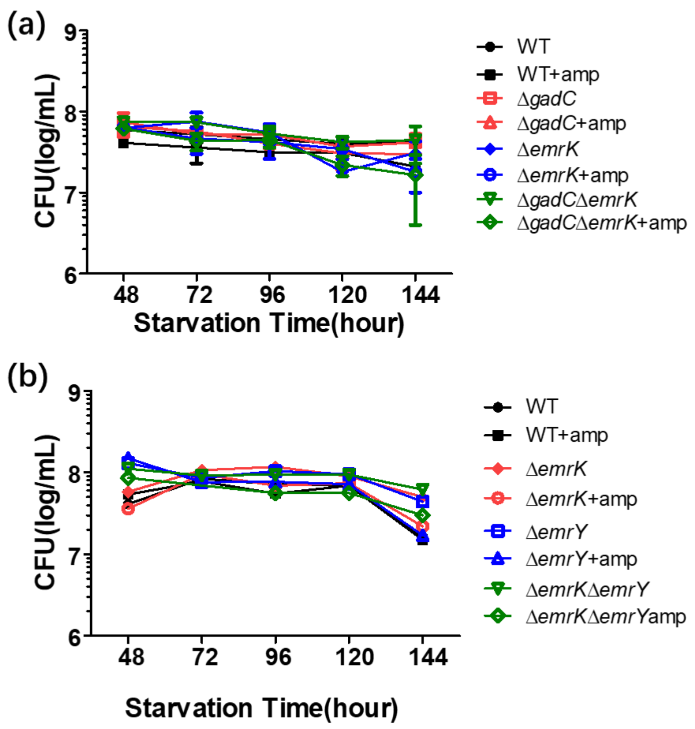


**Supplementary Figure S1. Ampicillin tolerance level of double knockout strains.**

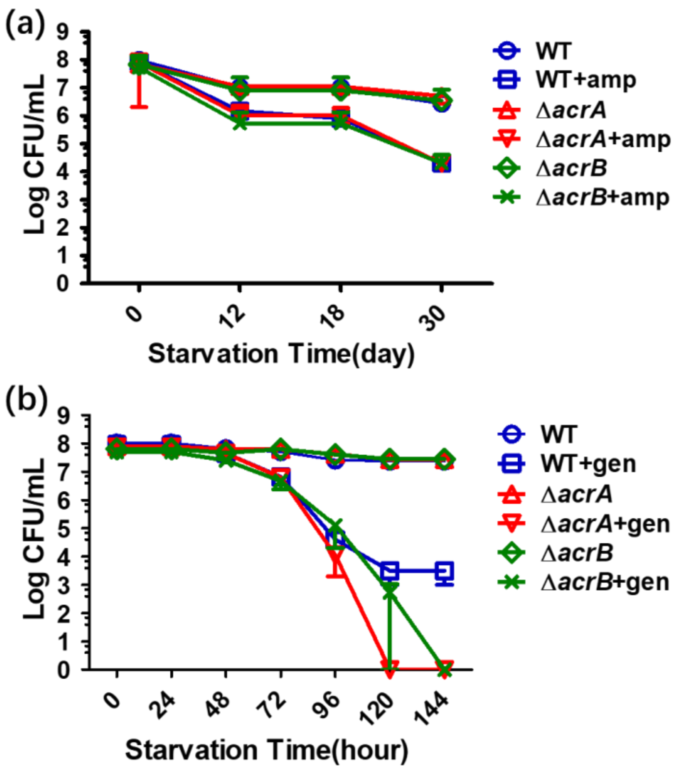


**Supplementary Figure S2. Starvation-induced tolerance to ampicillin and gentamicin in *acrA* and *acrB* gene knockout strains measured over a 1-month and six-days period, respectively.**
